# Normal Workflow and Key Strategies for Data Cleaning Toward Real-World Data: Viewpoint

**DOI:** 10.2196/44310

**Published:** 2023-09-21

**Authors:** Manping Guo, Yiming Wang, Qiaoning Yang, Rui Li, Yang Zhao, Chenfei Li, Mingbo Zhu, Yao Cui, Xin Jiang, Song Sheng, Qingna Li, Rui Gao

**Affiliations:** 1 Postdoctoral Research Station China Academy of Chinese Medical Sciences Beijing China; 2 Postdoctoral Works Station Yabao Pharmaceutical Group Co, Ltd Yuncheng China; 3 Xiyuan Hospital China Academy of Chinese Medical Sciences Beijing China; 4 First Affiliated Hospital Henan University of Traditional Chinese Medicine Zhenzhou China; 5 Beijing Xinshi Yuanzhi Technology Consulting Co, Ltd Beijing China

**Keywords:** data cleaning, data quality, key technologies, real-world data, viewpoint

## Abstract

With the rapid development of science, technology, and engineering, large amounts of data have been generated in many fields in the past 20 years. In the process of medical research, data are constantly generated, and large amounts of real-world data form a “data disaster.” Effective data analysis and mining are based on data availability and high data quality. The premise of high data quality is the need to clean the data. Data cleaning is the process of detecting and correcting “dirty data,” which is the basis of data analysis and management. Moreover, data cleaning is a common technology for improving data quality. However, the current literature on real-world research provides little guidance on how to efficiently and ethically set up and perform data cleaning. To address this issue, we proposed a data cleaning framework for real-world research, focusing on the 3 most common types of dirty data (duplicate, missing, and outlier data), and a normal workflow for data cleaning to serve as a reference for the application of such technologies in future studies. We also provided relevant suggestions for common problems in data cleaning.

## Introduction

Randomized controlled trials (RCTs) are considered to yield the highest-level evidence in the practice of evidence-based medicine, representing the “gold standard” for evaluating the safety and efficacy of drugs [1]. However, the extrapolation of RCT results to real-world situations is limited because of strict screening conditions, single-intervention measures, and limited sample sizes [2]. To compensate for the shortcomings of RCTs, Kaplan et al [3] first proposed the concept of real-world research (RWS) in 1993. RWS focuses on using high-quality, real-world data to generate reliable evidence regarding the effects of medical interventions in a real-world environment to complement the evidence generated from traditional RCTs.

The role of massive real-world data in academic and business environments has become increasingly significant [4]. To better understand the value of these data, data mining and analyses are required [5]. However, real-world data from medical practice are usually generated without the strict process controls applied in clinical trials. For example, when data are collected from multiple sources, such as different hospitals or hospital systems, the rules for integration may not be identical, leading to quality issues such as duplicate, missing, and outlier data. These “dirty data” are ubiquitous in RWS. Among them, the large amount of storage space required for duplicate data affects the efficiency of the database, and the inappropriate processing of missing data can result in the loss of a considerable amount of potentially useful information. Furthermore, inconsistent or incorrect outlier data can seriously affect the results of data analyses and key calculations, which may even provide incorrect directions for subsequent academic research, resulting in a loss of time, effort, and funding [6].

The China Center for Drug Evaluation issued a document titled “Guidance on Real-World Data for Generating Real-World Evidence (Trial)” [7] (referred to as “the guidance” in this study) in 2021, which emphasizes that “not all real-world data can produce real-world evidence when analyzed.” The role of data cleaning is to process dirty data to regenerate real-world data that can be used to form real-world evidence. A standardized data cleaning process is critical to improving data quality. The guidance proposes essential requirements for real-world data governance; however, it does not outline the specific processes and approaches for data cleaning in detail. In this study, we outlined the current data-cleaning approaches for RWS and proposed a normal workflow for data cleaning to serve as a reference for applying such technologies in future studies.

## Impact of Data Cleaning on Data Quality

Data cleaning is the process of identifying and solving problems, which is crucial for the management of data quality [8]. The lack of an effective data cleaning process may result in a “garbage in and garbage out” scenario [8], adversely affecting the subsequent data analysis. In contrast, an effective data-cleaning process can transform dirty data into clean, reliable data that reflect real-world situations, providing researchers with more valuable information [9]. Therefore, data cleaning plays a decisive role in improving data quality.

### Categorizing Issues With Data Quality

**Figure 1:**
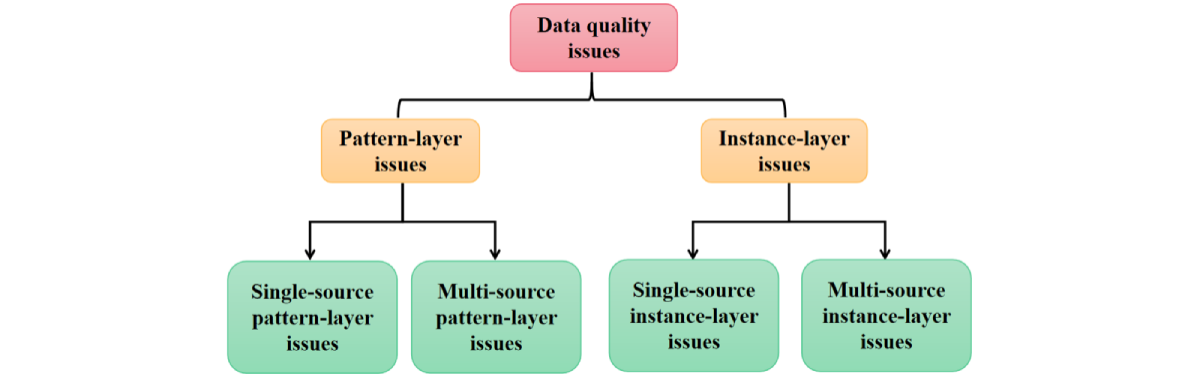
Classification of data quality issues.

Data quality is the degree to which the accuracy, completeness, consistency, and timeliness of the data satisfy the expected needs of specific users. Issues with data quality can be categorized as either pattern-layer or instance-layer, depending on the level at which the issues are observed. Similarly, issues can be categorized as single-source or multi-source, depending on the data source. Therefore, issues with data quality are typically divided into 4 categories: single-source pattern-layer issues, multi-source pattern-layer issues, single-source instance-layer issues, and multi-source instance-layer issues (Figure 1) [10].

### Causes of Data Quality Issues

Pattern-layer issues originate from deficiencies in the system design. For single data sources, pattern-layer issues include a lack of integrity constraints and low-end architectural design. For multiple data sources, pattern-layer issues can also include structural and naming conflicts among the sources. Pattern-layer issues are not the main focus of data governance for RWS; however, many issues in the instance layer are caused by unresolved errors in the pattern layer.

At the instance layer, data issues mainly arise from human errors, which is a key focus of RWS on data governance. Common causes of data record exceptions at the single-source instance layer include data input errors, similar or duplicate records, and missing values. Input errors occur mostly during the process of case recording and are common in data sources that rely heavily on manual input, such as hospital information system data and individual health monitoring data from mobile devices. Similar or duplicate records may arise from operational errors during manual data entry. However, they may also arise when 2 cases with different levels of completeness are stored for the same patient during the same time period. This latter scenario is common when exporting data for different time periods, such as from January to June and June to December successively. Missing values may arise from technical errors in recording or deliberate concealment on the part of the patient (eg, refusal to provide relevant information). Alternatively, missing values can be caused by failures in data storage or error clearance resulting from equipment issues. In some cases, highly sensitive data may also be difficult to obtain (eg, medical insurance payment data).

In addition to all the problems that can arise at the instance layer for single sources, unique multisource issues in the instance layer include inconsistent data time and aggregation. Among them, similar or duplicate records resulting from identifying the same content as different objects (ie, use of different expressions) are the main problems.

Data cleaning can effectively address issues at the instance layer. To improve data quality, this step should be integrated into the processing pattern layer.

## Data Cleaning

### Definition of Data Cleaning

Data cleaning is a series of processes that streamline the database to remove duplicate records and convert the remaining information into standard-compliant data. More specifically, data cleaning involves the preprocessing of the extracted raw data, which includes elements such as the removal of duplicate or redundant data, logical verification of variable values, treatment of outliers, and processing of missing data. Thus, any operation performed to improve data quality can be classified as data cleaning. Data cleaning also encompasses all processes used to detect and repair irregularities in data collection and improve data quality. In the process of data cleaning, the corresponding cleaning rules can be formulated, and the data-cleaning framework and cleaning algorithms can be used to make the data-cleaning process easier and more efficient.

The guidance suggests that real-world data can be obtained prospectively and retrospectively, requiring data management and governance, respectively. Data cleaning is an element of data governance and is not required in the data management process. Therefore, data cleaning is generally suitable for real-world data collected retrospectively. The guidance divides data cleaning in RWS into the processing of duplicate, outlier, and missing data.

### Basic Process for Data Cleaning

**Figure 2:**
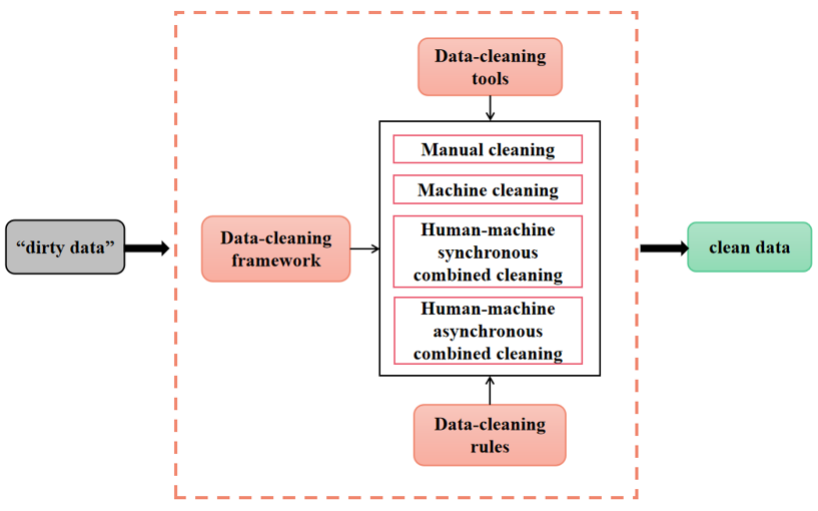
Basic process of data cleaning.

Relevant technical means, such as data mining, mathematical statistics, or predefined cleaning rules, are used to convert dirty data into data that meet quality requirements (Figure 2). Data cleaning is generally divided into 4 types: manual cleaning, machine cleaning, synchronous human-machine combined cleaning, and asynchronous human-machine combined cleaning [11]. The unprocessed source data are first collected directly from the database (ie, dirty data), following which the corresponding data cleaning rules are applied. The process can be streamlined using an appropriate data-cleaning framework and cleaning algorithms. Fully manual, fully automated, or combined strategies can be used until quality requirements are met.

Despite its high accuracy, manual cleaning is only suitable for smaller data sets, given its time-consuming nature. In contrast, machine cleaning is more suitable for processing larger data sets since the process is completely automated. However, the cleaning plan and program must still be developed in advance, making later-stage maintenance difficult. In the synchronous human-machine strategy, problems that cannot be handled by the machine are manually addressed through an appropriate interface. This method is advantageous because it reduces the workload of manual cleaning while reducing the difficulty of designing the machine cleaning strategy. In principle, the asynchronous human–machine strategy is similar to its synchronous counterpart; however, when problems that cannot be handled by the machine are encountered, the issues are not addressed in real time. Instead, a problem report is generated, and cleaning proceeds to the next step. Thus, manual processing occurs after cleaning and is the method currently used by most cleaning software.

## Normal Workflow for Data Cleaning

**Figure 3:**
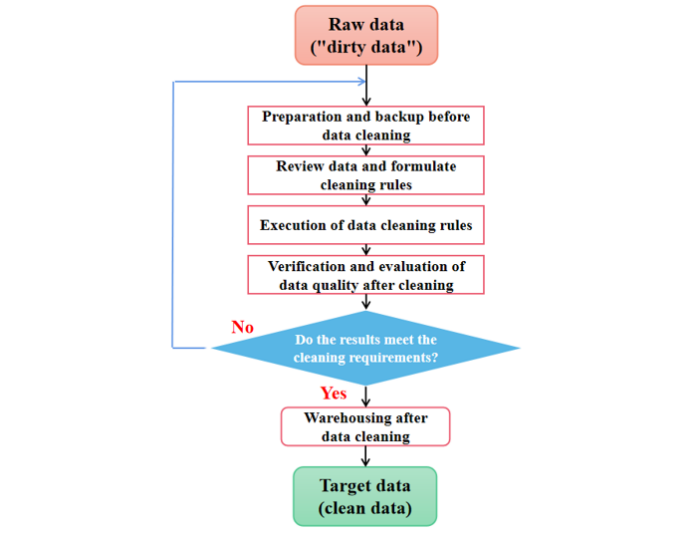
Normal workflow for data cleaning.

Depending on the task requirements, the data-cleaning workflow can be performed differently. The general data-cleaning process can be divided into 5 components (Figure 3).

### Step 1: Back Up and Prepare the Raw Data for Cleaning

Data collected from different sources must be combined before further data governance and analysis can be performed. Therefore, it is necessary to unify the data types, formats, and key variable names in different databases before data cleaning. In addition, the original data must be backed up and archived before cleaning to prevent damage or loss of data during the cleaning process. This step is also crucial in cases requiring cleaning policy changes.

### Step 2: Review the Data to Formulate Cleaning Rules

Appropriate cleaning methods (manual, machine, or combined) should be selected according to the size of the data set. After analyzing and summarizing the characteristics of the data source, the proposed cleaning algorithm and corresponding cleaning rules are formulated. Cleaning rules are divided into 3 categories: processing of missing, duplicate, and outlier data.

### Step 3: Implement the Cleaning Rules

**Figure 4:**
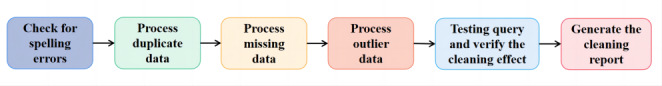
Execution of data cleaning rules: an example sequence.

The execution of cleaning rules is the core step in the data cleaning process, and data processing can be performed in the following order: duplicate, missing, and outlier data (Figure 4). However, given the differences in professional fields and situational factors, adopting a common, unified standard for data cleaning is difficult. In addition, there are many types of data quality problems and complex situations, making a generalization based on categories difficult. Therefore, the corresponding cleaning rules must be formulated on a situational basis.

### Step 4: Verify and Evaluate the Quality of the Cleaned Data

Following data cleaning, the quality of the data should be assessed according to the cleaning report generated, and problems that could not be addressed by the machine must be handled manually. Evaluating the data quality will also enable the optimization of the program and algorithm to ensure that future processes yield data of sufficient quality. After redesigning the program based on these observations, the cleaning step should be repeated as needed until the requirements for analysis have been met.

### Step 5: Warehouse After Data Cleaning

Following data cleaning, a new target database should be established for the cleaned data. While this aids in archiving and preservation, appropriate warehousing of the data can prevent the need for repeated cleaning work in the future.

## Summary of Data Cleaning Methods for the Instance Layer

This section describes the methodology of the data cleaning methods, including the data sets, the 3 types of dirty data, and the corresponding data cleaning methods.

### Data Set

In this study, we used a data set from a retrospective heart failure cohort in the Research Resource for Complex Physiologic Signals (PhysioNet) database [12]. This heart failure cohort retrospectively collected electronic medical records of 2008 hospitalized patients with heart failure from the Fourth People’s Hospital of Zigong City, Sichuan Province, China, from December 2016 to June 2019. The identification of hospitalized patients with heart failure was based on the International Classification of Diseases-9 code. Furthermore, the diagnostic criteria followed the 2016 European Society of Cardiology Heart Failure Diagnosis and Treatment Guidelines. The partial information contained in the data set is presented in Figure S1 in Multimedia Appendix 1, with 167 variables (n=167), including 2008 records (N=2008), and saved as a CSV file.

To provide a more intuitive demonstration of the results in the following examples, we added 30 records as “duplicate data” in the heart failure data set and manually adjusted the “systolic blood pressure” values in 11 records as “abnormal data.” According to the admission way (column E), patients with heart failure were divided into 2 groups: the emergency and nonemergency groups, and the urea values (column BO) were analyzed and compared between these 2 groups. There are “missing data” in the urea values of the 2 groups (Figure S2 in Multimedia Appendix 1):

### Processing of Duplicate Data

Methods for detecting duplicate data can be divided into record-based and field-based methods. Record-based duplicate detection algorithms include the N-grams, sorted-neighborhood method (SNM), clustering, and most probable number (MPN) algorithms [13-15]. Field-based repeat detection algorithms include the cosine similarity function [16] and Levenshtein distance algorithms [17]. The main processes involved in duplicate data cleaning are as follows:

Step 1: Analyze the attribute segments of the source database, limit the key search values of the attributes (eg, name, patient ID, and date of treatment), and sort the data in the source database from the bottom to the top or top to bottom according to the key search values.

Step 2: Scan all data records according to the order of arrangement, compare the adjacent data, and calculate the similarity of the matching data records. The duplicate data retrieval code for the sample data set is presented in Figure S3 in Multimedia Appendix 1.

Step 3: Deduplicate or merge the duplicate data. When the similarity value of adjacent data is higher than the threshold defined by the system, the continuous data records are identified as similar or duplicate data. The duplicate data retrieval results of the sample data set are presented in Figure S4 in Multimedia Appendix 1. These data should be deduplicated or merged. Similarly, when the similarity value is below the threshold defined by the system, scanning should be continued, and steps 2 and 3 should be repeated as necessary.

**Figure 5:**
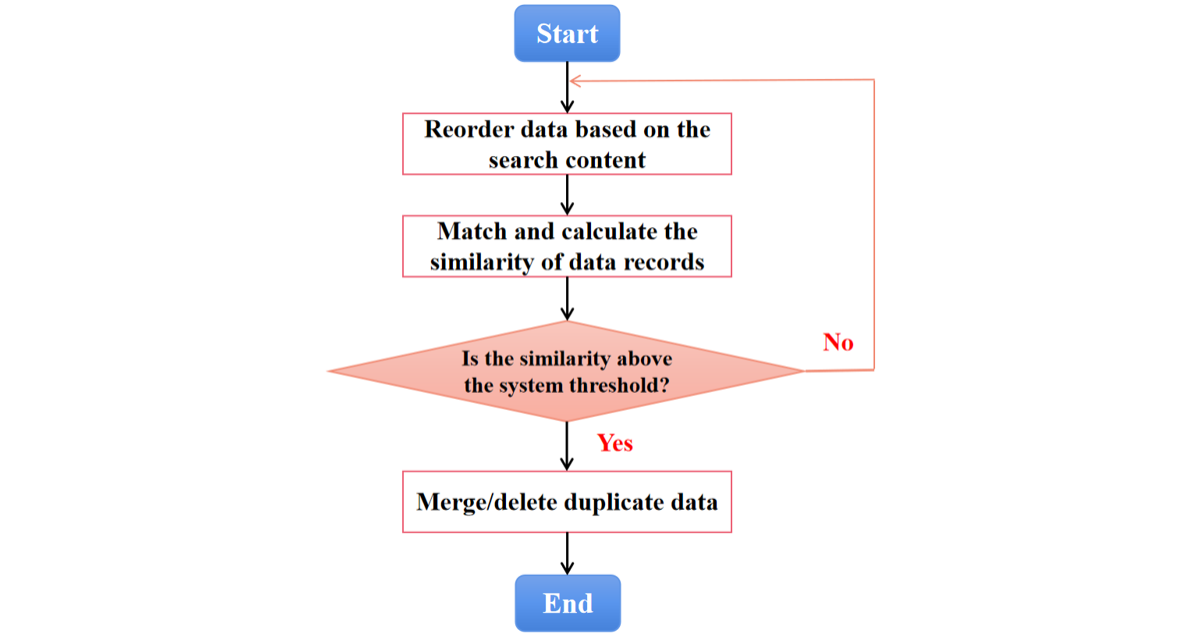
Cleaning workflow for duplicate data.

Step 4: After testing all data records, generate a report and archive the data before and after it. The workflow for cleaning duplicate data is shown in Figure 5.

### Processing of Missing Data

While deletion is initially considered for missing values, this only applies if the data set is large, and the proportion of missing data is not large. If the amount of data is not sufficiently large, directly deleting missing values will lead to data loss, resulting in the deletion of many useful statistics. Feasible methods for addressing missing values include using missing-value imputation technology for repair. Commonly used methods for imputation include mean imputation, mode imputation, minimum imputation, regression imputation, and maximum likelihood estimation [18-21], and using a Bayesian or decision tree classifier [22] to model the missing value imputation as a classification problem. The main processes involved in cleaning missing data are as follows:

Step 1: Perform parameter estimation for missing values in the source data and select the deletion method or imputation method according to the proportion of missing values. The missing data retrieval code for the sample data set is presented in Figure S5 in Multimedia Appendix 1.

Step 2: Fill in the missing data according to the data-filling algorithm. The missing data retrieval and interpolation results of the sample data set are presented in Figure S6 in Multimedia Appendix 1. For the convenience of demonstration, the mean interpolation method was chosen, and specific problems should be analyzed in practical application.

**Figure 6:**
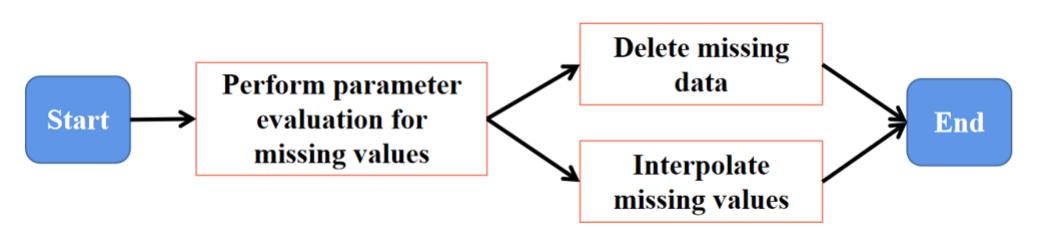
Cleaning workflow for missing data.

Step 3: Output and archive the complete data. The workflow for cleaning missing data is shown in Figure 6.

### Processing of Outlier Data

An outlier is a value that does not conform to attribute semantics. There are 2 methods for handling outlier data: deletion and replacement. However, the appropriate methods should be selected based on the nature of the data. If the nature of the outlier data is unsuitable for replacement, such as age data, outlier analysis can be used to detect and delete outliers. Outlier detection algorithms mainly include cluster-based, statistical model-based, density-based, and proximity-based algorithms [23]. If the nature of the abnormal data is suitable for replacement, the regression method or mean smoothing method can be used to replace the abnormal values. The regression method is applicable to data conforming to a linear trend, while the mean smoothing method is more effective in cleaning data with sinusoidal time-series characteristics [24]. The main processes involved in cleaning outlier data are as follows:

Step 1: Convert the source data into the data format required for detection and conduct data preprocessing. The exception data retrieval code for the sample data set is presented in Figure S7 in Multimedia Appendix 1.

Step 2: Perform outlier detection on the data after preprocessing. The abnormal data retrieval results of the sample data set are presented in Figures S8 and S9 in Multimedia Appendix 1. If the nature of the outlier data is not suitable for replacement, delete the outliers. If the nature of the outlier data is suitable for replacement, use the regression or mean smoothing method to replace the outliers. Often, the repaired data can lead to new data exceptions, making it necessary to repeat steps 1 and 2 until the requirements are met.

**Figure 7:**
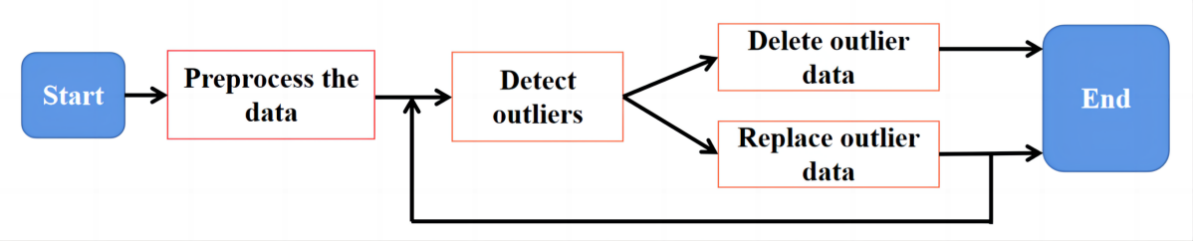
Cleaning workflow for outlier data.

Step 3: Restore the repaired data to its original format and perform archiving. The workflow for cleaning outlier data is shown in Figure 7.

## Data Cleaning Tools

Oni et al [25] reported 4 tools commonly used in the data cleaning industry: Data Wrangler, OpenRefine, Python, and R. They explained that these tools are the most popular tools for data cleaning in RWS. OpenRefine, R, and Python are all open-source tools, making them easy to access and use. Data Wrangler is a commercial tool, but there is a community version that efficiently cleans up data. The characteristics of these tools are described below and presented in Table 1.

**Table 1:** Comparison of Data Wrangler, Python, R, and OpenRefine.

Criteria	Data Wrangler	Python	R	OpenRefine
Import format	Excel, CSV, and text	All	All	Excel, CSV, TSV^a^, XML, JSON, and RDF^b^
Factors affecting the performance time	Data size and user choice	User programming skill level	User programming skill level	Data size and data format
Output format	CSV, JSON, and TDE^c^	Any format	Any format	Excel, TSV, CSV, and HTML table
Skill level	Basic level	Advanced level	Advanced level	Basic or intermediate level
Running platform	Windows and Mac	All	All	All
Accuracy	Depends on the specific data quality issues (eg, missing values)	Depends on the user’s programming skill level	Depends on the user’s programming skill level	Depends on the specific data quality issues (eg, missing values)
Possibility to embedded	No	Yes	Yes	No, but code is available
Data set processing scale	Big data	Big data	Big data	Up to 5000 records
Graphic user interface	Yes	No	No	Yes

^a^TSV: tab separated value.

^b^RDF: resource description framework.

^c^TDE: tableau data extract.

### Data Wrangler

Data Wrangler is a web-based data cleaning and reorganization project developed by Stanford University [26]. It is a web-based data cleaning tool, mainly used to remove invalid data and organize data into user-required formats. Several data collations can be done in Data Wrangler with a simple click. It also lists the history of data modifications, making it extremely convenient for users to view past modifications and undo a modification operation. Data Wrangler can process data in 2 ways: users can either paste the data into its web interface or use the web interface to export any data operations to Python code and process them.

Advantages of Data Wrangler are that it has column and grid views, uses natural language to describe transformations, supports data visualization and every step of data cleaning, and supports large-scale editing. Disadvantages are that the free version of Data Wrangler provides only limited functionality and consumes a large amount of memory.

### Python

Python is a concise, easy-to-read, and extensible data-cleaning tool [27]. Currently, Numpy and PANDAS (Python Data Analysis Library) are the most used mainstream modules in Python for data cleaning. The PANDAS module is mainly used for data analysis, of which data cleaning is a part. The Numpy module has a powerful N-dimensional array object, and vectorization operations make data processing efficient and helpful in cleaning large data sets.

Advantages of Python are that it is easy to embed into other tools and applications, and users can customize solutions based on their needs. Disadvantages are that it requires users to have advanced programming skills, learn how to use many modules in Python, and understand the required steps during the cleaning process in advance, making it difficult to implement.

### R

R is the language and operating environment used for statistical calculations, data analysis, and graphics [28]. R is a free, open-source software belonging to the GNU’s Not Unix (GNU) system. It can provide some integrated statistical tools. More importantly, it can provide various mathematical and statistical calculation functions, allowing users to flexibly analyze data and create new statistical calculation methods that meet their needs. R has a set of tools that can effectively and comprehensively clean data. The R environment can read data in multiple formats and process these files. R provides sufficient visualization tools. During the cleaning process, visualization of data at each stage is useful.

Advantages of R are that it supports the visualization of data and each step of data cleaning, making it more suitable for analyzing statistical data. Disadvantages of R are that it is not a good choice for projects outside data science. Users must understand the required steps during the cleaning process in advance, making it difficult to implement.

### OpenRefine

OpenRefine is a web-based ,independent, open-source application with various functions such as data portrait, cleaning, and conversion [29]. It can perform visual manipulations on data. It is similar to traditional Microsoft Excel software. However, it works like a database, as it does not deal with individual cells but rather with columns and fields. OpenRefine, formerly known as Google Refine, is a tool for cleaning, reshaping, and editing bulk, unstructured, and cluttered data. OpenRefine is a desktop application that opens as a local web server in a browser. Since it is an open-source project, its code can be reused in other projects. OpenRefine performs cleanup tasks by filtering and faceting, and then converts the data into a more structured format.

Advantages of OpenRefine are that it is a desktop application that does not require networking, making data sets more difficult to tamper with and relatively secure. It can be easily operated and has powerful functions for converting data. Users can use its facet function to filter data into subsets. Disadvantages include a limit of 5000 records, making OpenRefine not suitable for processing large data sets. It assumes that data is organized in a tabular format with limited operations and an unfriendly user interface. In addition, Google has removed support for the tool.

## Documentation and Reporting

The Guidelines for Real-World Evidence to Support Drug Research and Development and Review (Trial) of the National Medical Products Administration of China (No 1 of 2020) stipulated that “transparency and reproducibility of evidence” should be achieved in the process of translating real-world data into real-world evidence, noting that proper documentation retention is the basis for ensuring transparency and reproducibility. We recommend that the data cleaning plan be stipulated in the RWS data governance plan, which should include personnel requirements, previous expectations for screening suspicious data, diagnostic procedures for identifying errors in the source data, cleaning tools, and decision rules to be applied in the cleaning phase.

Additionally, appropriate documentation should be provided at each of the following points: (1) precleaning (the raw data stage); (2) cleaning operation (during this stage, documentation should include differential markers of suspicious feature types, diagnostic information related to the type of dirty data, application algorithms and operational steps for data editing, and corresponding cleaning reports generated after cleaning is complete; simultaneously, the modification date must be marked for each operation, and the information of the relevant personnel involved in the modification must be saved); (3) the retention stage (after cleaning the data).

## Recommendations for Data Cleaning

Most research projects do not formulate data-cleaning plans in advance. Analyses performed without complete cleaning of the dirty data will lead to biased results, and identifying the causes of any deviations from scratch will further delay the progress of the work. As the diversity of data sources increases the difficulty and workload of data cleaning, we recommend the following strategy.

First, formulate the cleaning plan in advance. As mentioned above, the results of statistical analyses are closely related to the cleanliness of the data. Data cleaning plans should be formulated in advance to ensure sufficient time and technical guidance for data cleaning.

Second, cultivate medical and computer talent. While analyzing real-world data, many medical researchers find that they do not understand computer programming. Conversely, many computer programmers do not have much medical expertise, resulting in poor communication between the two sides and affecting the development of data-cleaning strategies. Therefore, it is necessary to cultivate a group with compound talents who understand both medical statistics and computer applications.

Third, strengthen the computer skills training required for data cleaning. Hospitals and data companies should work together to organize and implement skills training for data cleaning in a timely manner. In addition, medical researchers and computer programmers should participate simultaneously to acquire professional knowledge from each other. Machine-based and manual methods can be selected to improve work efficiency when adopting combined human-machine cleaning strategies.

Fourth, establish a unified data governance and management platform. Researchers should fully use modern technical means to realize the collection, review, governance, and management of RWS data. Moreover, project researchers should perform unified management and maintenance of platform data.

## Conclusions

Real-world data are large-scale with low value density. The data source yields dirty data, plagued by issues such as duplication, missing values, and outliers owing to various reasons. Analyses based on such data can severely reduce the efficiency of data use and negatively affect the quality of decision-making. Data cleaning technology can improve data quality and provides more accurate and realistic target data than the source data, which can then be used to support data consumers in making appropriate decisions. The data cleaning principles and workflows discussed in this study may aid in developing standardized methods for data cleaning in RWS.

## Appendix

Multimedia Appendix 1 Supplementary figures 1-9.

## Abbreviations

GNU: GNU’s Not Unix
MPN: most probable number
PANDAS: Python Data Analysis Library
RCT: randomized controlled trial
RDF: resource description framework
RWS: real-world research
SNM: sorted-neighborhood method
TDE: tableau data extract
TSV: tab separated value

## References

[1] Bhide A, Shah PS, Acharya G (2018). A simplified guide to randomized controlled trials. Acta Obstet Gynecol Scand.

[2] Bothwell LE, Podolsky SH (2016). The emergence of the randomized, controlled trial. N Engl J Med.

[3] Kaplan NM, Sproul LE, Mulcahy WS (1993). Large prospective study of ramipril in patients with hypertension. CARE investigators. Clin Ther.

[4] Schalk E, Hentrich M (2022). Real-world data. Dtsch Arztebl Int.

[5] Corrigan-Curay J, Sacks L, Woodcock J (2018). Real-world evidence and real-world data for evaluating drug safety and effectiveness. JAMA.

[6] Page L (2005). The high cost of dirty data. Mater Manag Health Care.

[7] (2021). The guidance on real-world data for the generation of real-world evidence (Trial). The China Center for Drug Evaluation.

[8] Benevento E, Aloini D, van der Aalst WMP (2022). How can interactive process discovery address data quality issues in real business settings? Evidence from a case study in healthcare. J Biomed Inform.

[9] Bogani R, Theodorou A, Arnaboldi L, Wortham RH (2022). Garbage in, toxic data out: a proposal for ethical artificial intelligence sustainability impact statements. AI Ethics.

[10] Rahm E, Do HH (2000). Data cleaning: problems and current approaches. IEEE Data Eng.

[11] Gesicho MB, Were MC, Babic A (2020). Data cleaning process for HIV-indicator data extracted from DHIS2 national reporting system: a case study of Kenya. BMC Med Inform Decis Mak.

[12] Zhang Z, Cao L, Zhao Y (2000). Hospitalized patients with heart failure: integrating electronic healthcare records and external outcome data. PhysioNet.

[13] Nawab RMA, Stevenson M, Clough P (2014). Comparing medline citations using modified N-grams. J Am Med Inform Assoc.

[14] van Wunnik BPW, Visschers RGJ, van Asselt ADI, Baeten CGMI (2012). Cost-effectiveness analysis of sacral neuromodulation for faecal incontinence in the Netherlands. Colorectal Dis.

[15] Jahan M, Hasan M (2021). A robust fuzzy approach for gene expression data clustering. Soft Comput.

[16] Akbaş CE, Günay O, Taşdemir K, Çetin AE (2017). Energy efficient cosine similarity measures according to a convex cost function. Signal Image Video Process.

[17] Berger B, Waterman MS, Yu YW (2021). Levenshtein distance, sequence comparison and biological database search. IEEE Trans Inf Theory.

[18] Raja PS, Thangavel KJSC (2020). Missing value imputation using unsupervised machine learning techniques. Soft Comput.

[19] Silva-Ramírez EL, Pino-Mejías R, López-Coello M (2015). Single imputation with multilayer perceptron and multiple imputation combining multilayer perceptron and k-nearest neighbours for monotone patterns. Applied Soft Computing.

[20] Beesley LJ, Bondarenko I, Elliot MR, Kurian AW, Katz SJ, Taylor JM (2021). Multiple imputation with missing data indicators. Stat Methods Med Res.

[21] Li M, Liu X (2020). Maximum likelihood least squares based iterative estimation for a class of bilinear systems using the data filtering technique. Int J Control Autom Syst.

[22] Duan Z, Wang L, Sun M (2020). Efficient heuristics for learning Bayesian network from labeled and unlabeled data. Intell Data Anal.

[23] Shao M, Qi D, Xue H (2021). Big data outlier detection model based on improved density peak algorithm. J Intell Fuzzy Syst.

[24] Yang J, Zhu H, Choi T, Cox DD (2016). Smoothing and mean-covariance estimation of functional data with a bayesian hierarchical model. Bayesian Anal.

[25] Oni S, Chen Z, Hoban S, Jademi O (2019). A comparative study of data cleaning tools. Int J Data Warehous.

[26] Kandel S, Paepcke A, Hellerstein J, Heer J (2011). Wrangler: interactive visual specification of data transformation scripts.

[27] Raschka S, Patterson J, Nolet C (2020). Machine learning in python: main developments and technology trends in data science, machine learning, and artificial intelligence. Information.

[28] Fox J, Leanage A (2016). R and the journal of statistical software. J Stat Soft.

[29] Carlson S, Seely A (2017). Using OpenRefine's reconciliation to validate local authority headings. Cat Classif Q.

