# Supplementary material for: Normal Workflow and Key Strategies for Data Cleaning Toward Real-World Data: Viewpoint
Source: Interact J Med Res. 2023 Sep 21;12:e44310. doi: 10.2196/44310 (PMC10557005; doi:10.2196/44310)

**Figure S1**. Screenshot of heart failure.csv opened with Microsoft Excel.


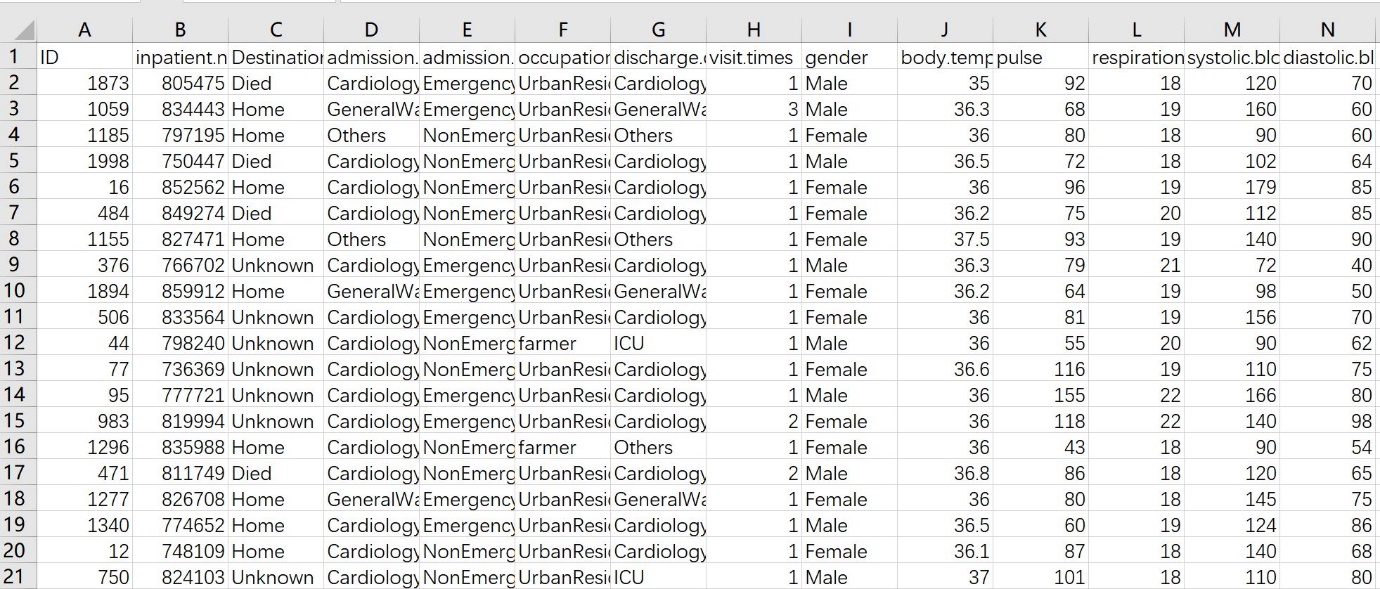


**Figure S2**. Demonstration interface for processing missing data information.


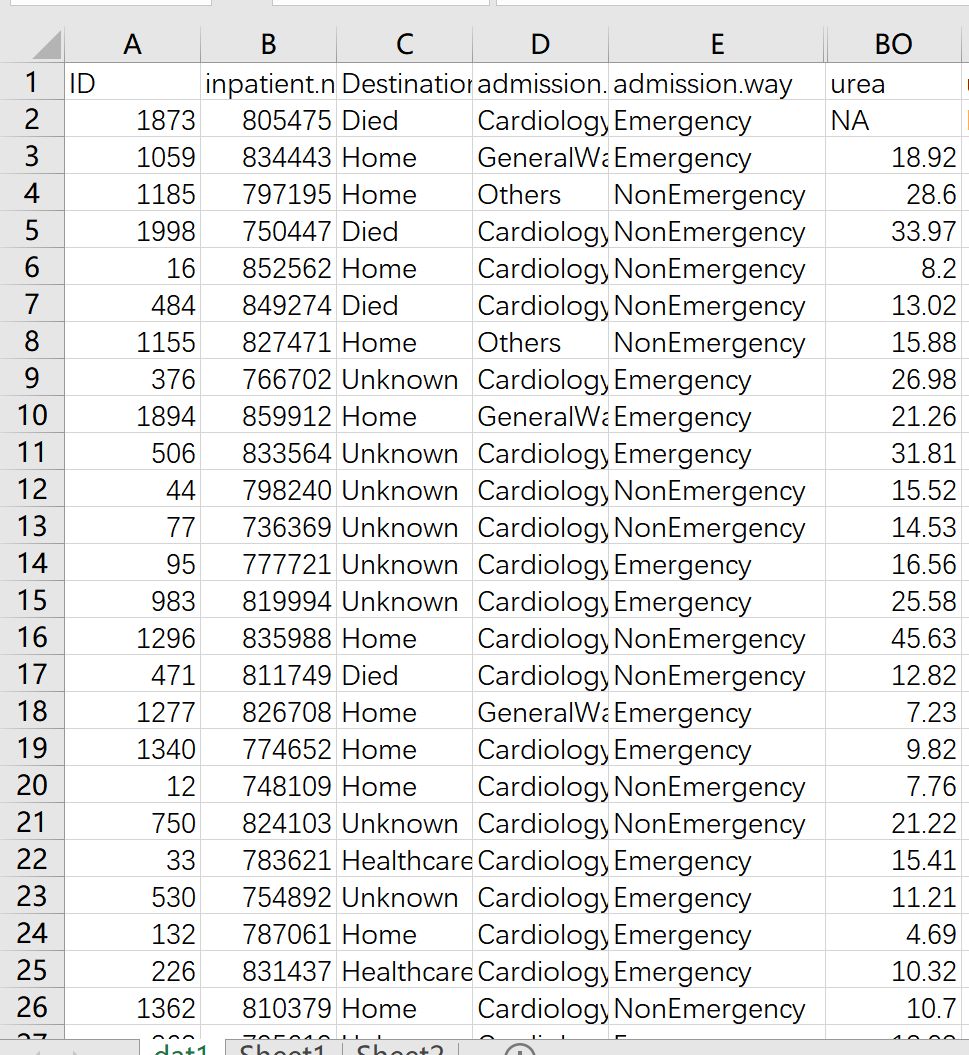


**Figure S3**. Retrieval code for duplicate data.


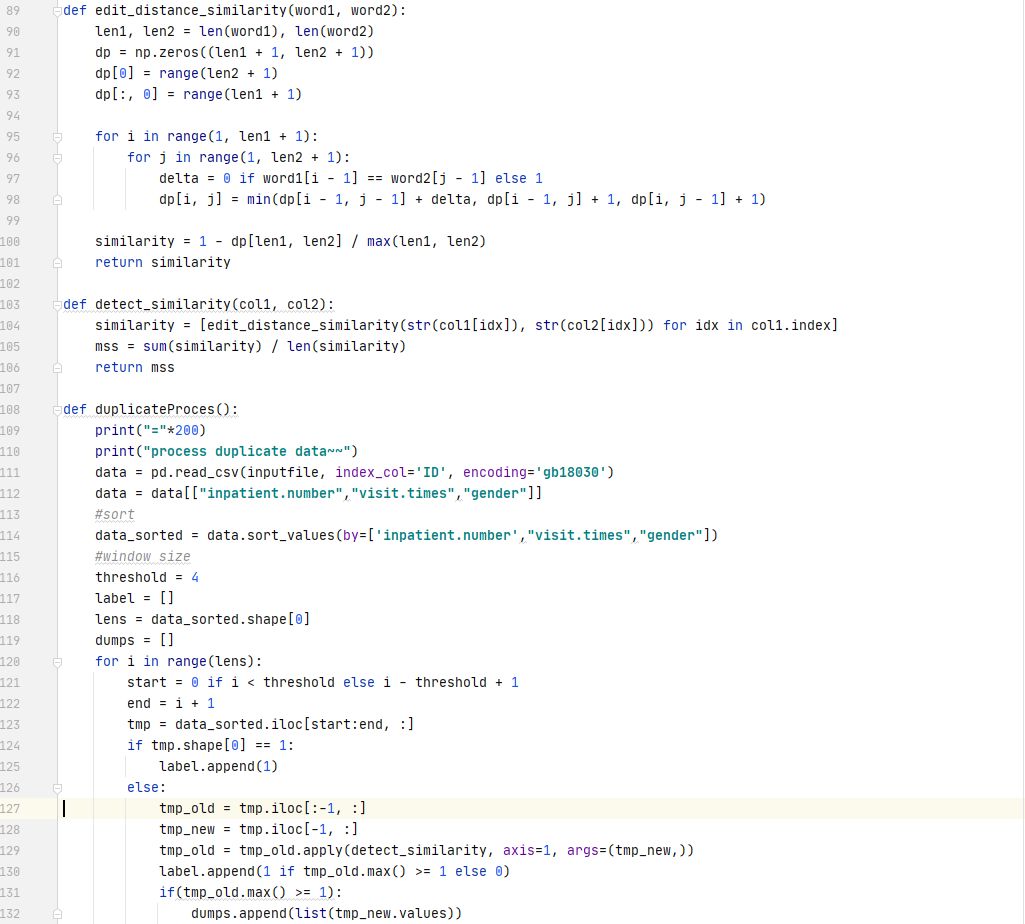


**Figure S4**. Retrieval results of duplicate data.


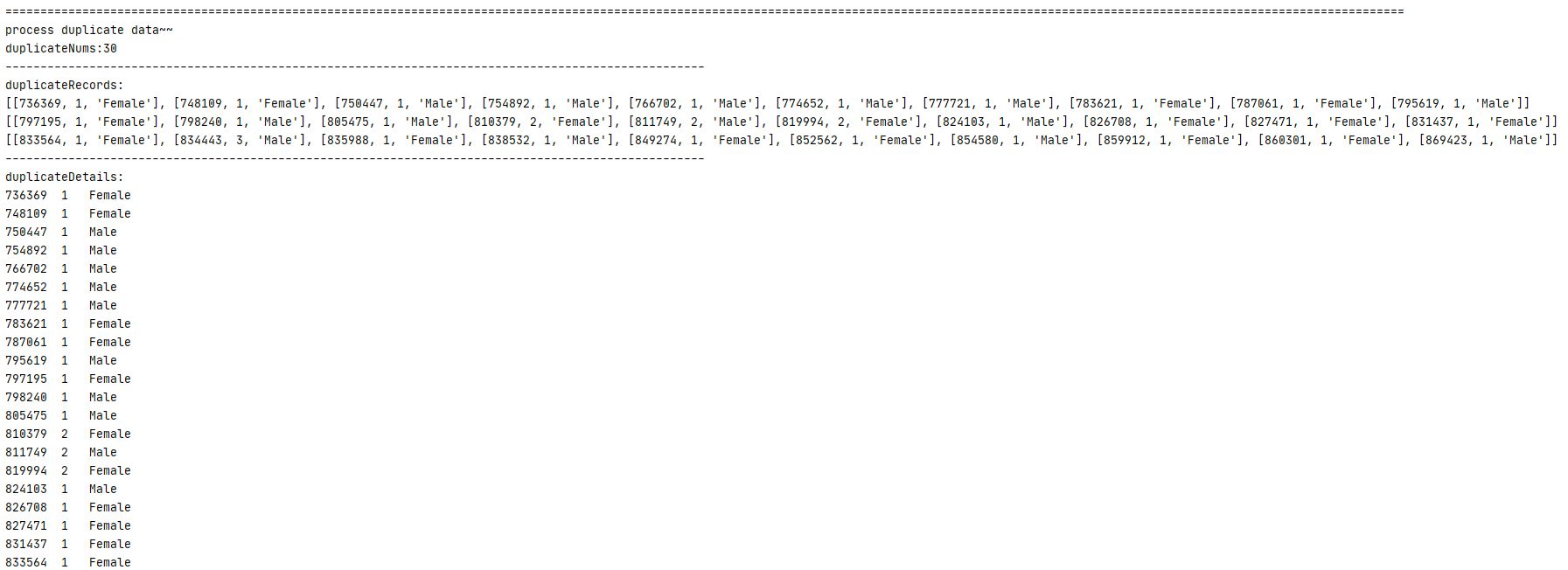


**Figure S5**. Retrieval code for missing data.


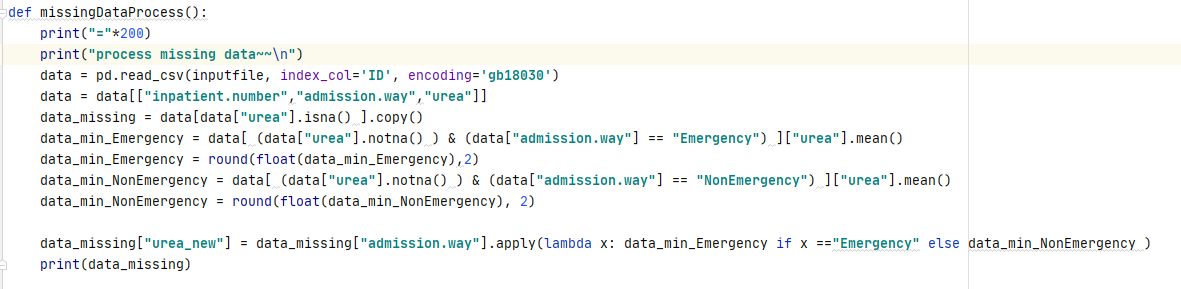


**Figure S6**. Retrieval and imputation results of missing data.


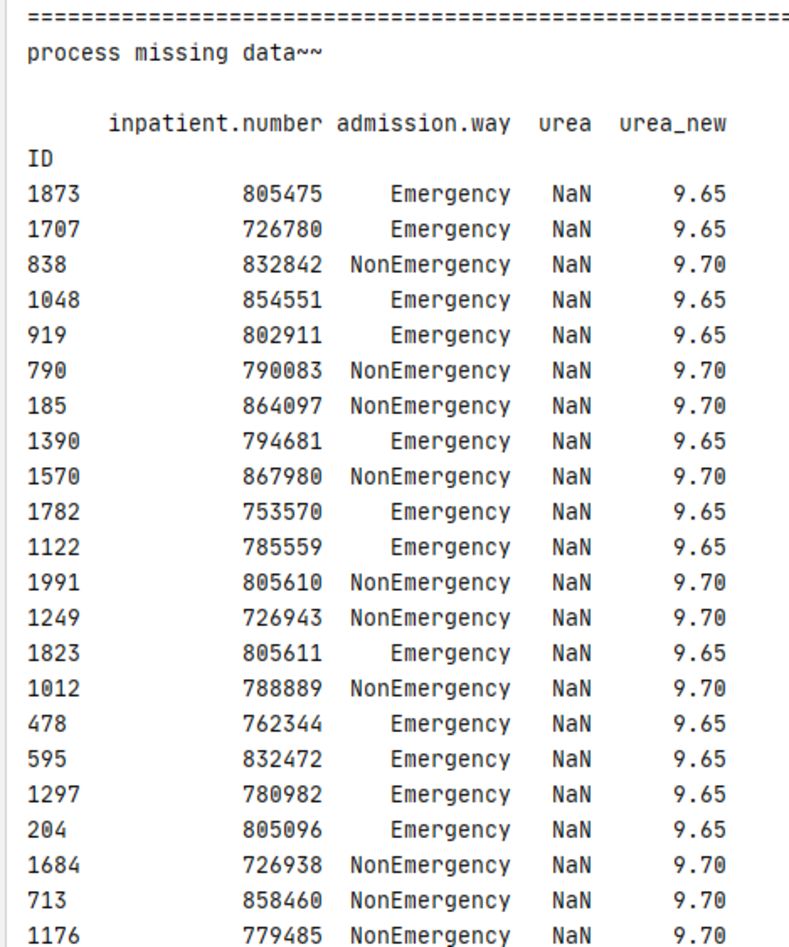


**Figure S7.** Retrieval code for abnormal data.


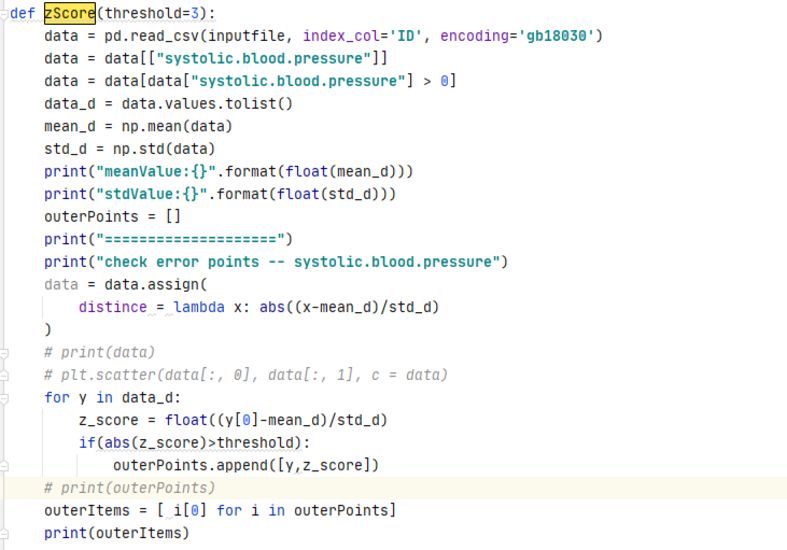


**Figure S8.** Retrieval results of abnormal data.


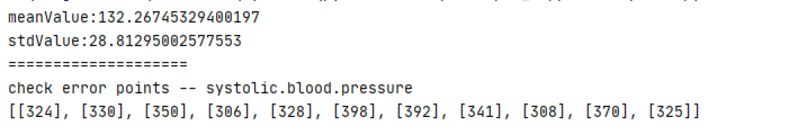


**Supplementary Figure 9**. Outlier scatter plot of abnormal data.


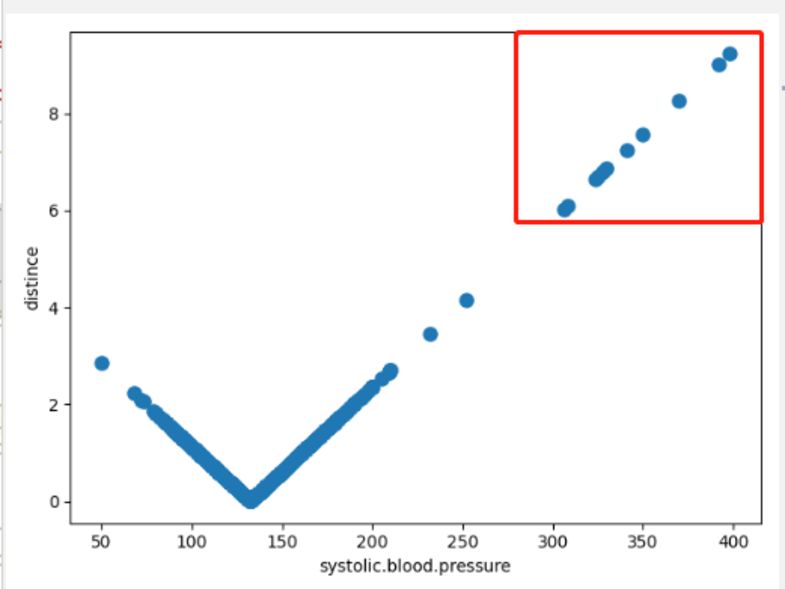

Supplement: Multimedia Appendix 1 [file ijmr_v12i1e44310_app1.docx]
